# Strategically creating maximally heterogeneous lab groups did not improve group performance in an introductory biology lab class

**DOI:** 10.1371/journal.pone.0323799

**Published:** 2025-05-15

**Authors:** Anisha S. Navlekar, Elli J. Theobald, Ken Griffith, Lisa B. Limeri

**Affiliations:** 1 Department of Biological Sciences, Texas Tech University, Lubbock, Texas, United States of America; 2 Department of Biology, University of Washington, Seattle, Washington, United States of America; 3 Teaching, Learning, and Professional Development Center, Texas Tech University, Lubbock, Texas, United States of America; Medical College of Wisconsin - Central Wisconsin Campus, UNITED STATES OF AMERICA

## Abstract

Collaboration is a critical skill for professionals in any field to master, and group work is a prominent component of many lab courses. However, there is conflicting guidance about the best method for forming groups to maximize performance and student experiences. Based on the benefits of cognitive diversity, we hypothesized that creating maximally heterogeneous groups would improve performance on lab activities. We conducted a quasi-experiment in the lab sections of a large-enrollment 2-semester introductory biology for majors course sequence (n = 986). In these large enrollment courses, students simultaneously enroll in smaller-enrollment lab sections. Each semester, we assigned groups randomly in half of the lab sections and in the other half of lab sections we strategically assigned groups to be maximally heterogeneous in terms of race, gender, and prior preparation. We examined the impact of group assignment on students’ academic performance (their grade on their collaborative lab report and their overall lab grade), incidence of group conflict, and student attitudes towards group work (i.e., teamwork satisfaction and perceptions of collaborative learning). We found that group formation strategy had no impact on students’ grades on either their collaborative lab report or their overall lab grade. Group conflicts were reported so infrequently that we were not able to detect any differences between the two groups. Our measures of groupwork satisfaction and perceptions of collaborative learning failed to demonstrate measurement invariance between the two types of group formation, which prevented us from assessing whether student attitudes differ, but suggest that there is some experiential difference that we were unable to capture.

## Introduction

The ability to collaborate with others and work well as a group member is a highly desirable skill for professionals in all fields. Diverse representation in the U.S. workforce along with globalization and interdisciplinary nature of these professional fields has necessitated employing such teamwork skills to generate solutions to society’s most challenging problems. Building collaboration skills is a worthy educational goal such that National Science Foundation’s Vision and Change initiative includes the “ability to communicate and collaborate with other disciplines” as a core competency [1]. Thus, it is imperative that undergraduate students who eventually enter this dynamic workforce must be trained and equipped to handle interactions and challenges associated with working in a team. One of the main ways for individuals pursuing careers in scientific fields to practice teamwork skills and collaboration is through coursework in their undergraduate degree. The laboratory component of STEM courses is an ideal setting to practice collaboration.

### Group work in undergraduate STEM classes

Group work is a key component of several different evidence-based pedagogical strategies, including course-based undergraduate research experiences [2], peer instruction [3], many active learning strategies, such as think-pair-share [4], and constructivist approaches in which students construct their understanding through discussion and problem-solving with groups [5]. Incorporating group work into classes can yield a variety of benefits for students. Multiple meta-analyses of group work, including one specific to STEM undergraduate courses, have concluded that group work tends to boost a number of desirable outcomes, including achievement, productivity, attitudes towards learning, and psychological health in students [6,7]. In STEM courses like biology, group work is particularly common and amenable in laboratory-based classes [8]. Lab courses that employ inquiry-based activities can simulate how scientists work and collaborate in real life in addition to training students in science process skills. This is best demonstrated and practiced when undergraduates work in groups in the lab.

Despite its numerous benefits, group work can be challenging to implement effectively and may also cause undesirable outcomes. Students dislike group work when they have group mates who show up to class late, are unprepared, or are not engaged in the group work [9]. Social loafing is a common group work challenge, in which some students take on the majority of the work while others contribute little to the group project [10]. Due to these and other challenges, many students have negative perceptions of group-based projects [11]. In an attempt to provide practical guidance to instructors, researchers have investigated how the way groups are formed and group compositions impact the effectiveness of group work and student outcomes.

### Group formation and composition

There is a great variance in the results of research on the best way to form groups or the ideal group composition. Generally, groups may be formed by students self-assorting or by instructor assignment. Self-assortment tends to result in more homogenous groups, since people generally tend to be more comfortable around and attract to others like themselves [12,13, but see 14]. There are multiple axes of heterogeneity that the literature unpacks: cognitive heterogeneity and demographic heterogeneity. Below we explore both.

When student choice factors into group selection, students could experience higher satisfaction in completing tasks, mainly due to increased comfort among group members leading to increased cohesiveness of the group. Theobald and colleagues showed that when students had a friend in their group, they were more comfortable, and this increased students’ content mastery by 27.5% [15]. This lends support to the idea that positive interdependence is one of the factors that impacted students’ experiences with group activities. Chapman and colleagues found that for first-year business students, self-selected groups perceived themselves to have better communication, more enthusiasm for group work, and a positive outlook towards their course outcomes [16].

However, the self-selection of students into groups can lead to a phenomenon of “cronyism.” Chapman and colleagues describe this as a situation where groups are based on prior relationships and familiarity and can cause cultural divide among students between groups [16]. Self-selection of groups is also shown to affect student achievement and lead to poorer attitudes towards learning [17,18]. Donovan and colleagues also observed that when asked to self-select, certain students reported feelings of anxiety when they could not find a group and ultimately approached the instructor for group assignment [14]. Thus, allowing students to self-assort into groups could pose challenges to creating inclusive and equitable learning environments for marginalized students if their peers do not seek them out for inclusion in their groups.

An alternative that potentially relieves stress on students to find a group they fit in is for instructors choose to assign groups. However, instructors then must decide how to create these assignments. The ideal composition of groups remains elusive as literature contains mixed results (see Table 1 in [14]). Creating diverse groups is an appealing approach because heterogeneity in perspectives and ways of thinking can boost overall group performance in problem solving [19,20]. Donovan and colleagues examined student performance in heterogeneous groups in a large-enrollment nonmajors biology course and found that group work improved learning outcomes of students who scored low on a biology competence assessment and did not impair the performance students who scored intermediate and high [14]. Students of all ability levels had more positive views of group work in heterogenous groups compared to students in more homogenous groups [14].

**Table 1:** Student demographics.

Demographic	Number of students	%
**Gender**		
Men	270	33.37
Women Non-binary	530 9	65.51 1.11
**Generation** ^1^		
First generation	265	33.08
Continuing generation	536	66.92
**Class Standing**		
First year	87	10.77
Second year	431	53.34
Third year	219	27.10
Fourth year	71	8.79
**Major**		
Life Sciences	582	77.19
Not Life Sciences	172	22.81
**Race/Ethnicity**		
American Indian or Alaska Native	13	1.44
Asian	119	13.15
Black or African American	69	7.62
Hispanic or Latino	268	29.61
White	436	48.18

^1^First generation was defined as having no parents or guardians who have earned a 4-year college degree.

Student demographics from both semesters as self-reported by students on end of semester surveys. Students could opt out of completing the survey or skip questions and could select multiple identities for race/ethnicity, so counts may not sum to the total number of students enrolled over the academic year (n = 986 unique students). If a student was enrolled in both semesters, their demographics are reported only once. Patterns were similar for the two semesters so the data are shown in aggregate here.

In addition to cognitive heterogeneity, research on group composition has considered the role of demographic heterogeneity. A survey of over 1,000 students from 6 different institutions found that cultural diversity promoted behavioral and cognitive engagement [21]. Similarly, a large-scale study of over 11,000 undergraduates at 20 institutions found that courses with higher representation of first-generation and racially marginalized students had higher average grades for the entire class [22]. Samudra and colleagues suggest that student groups with diverse representation tend to interact with each other more and thus transfer information and knowledge among themselves, leading to opportunities of peer learning [9]. This effect has also been found on a smaller scale by comparing groups within a STEM class; Donovan and colleagues found that demographically diverse groups have had higher learning outcomes than self-selected, more homogenous groups [14]. Rienties and colleagues showed a similar outcome in their study with students in an event management course and observed that although students in groups with demographically diverse members initially faced cultural differences, they were able to overcome them using coping strategies, leading to a stronger team identity [23]. Working in heterogenous groups can yield career-lasting impacts for professional scientists; Campbell and colleagues showed that journal articles in ecological science published by groups with gender diversity (at least one man and woman on the team) were 34% more likely to be cited than those published by gender-uniform groups [24]. This trend was repeated in journal articles published in the medical sciences [25] and in innovation process teams in management organization [26]. All these studies emphasize not only the real-world application of teamwork, but also the value of heterogeneity among team members.

Despite the numerous documented benefits, working in both cognitively and demographically heterogenous groups also poses challenges. When groups are heterogeneous in members’ identities, there is potential for individuals belonging to underrepresented and marginalized groups to experience feelings of marginalization and be subjected to bias and discrimination by their group mates [27]. Studies find that students with minoritized identities or those facing stereotype threats are more likely to be reluctant and passive while participating in a group, especially if they are outnumbered by members with majority identities [28,29]. For example, Adams and colleagues found that women tended to decrease their participation in a group that contained more men [28]. Another study with students in an introductory physics lab course indicated that task division in heterogenous groups became gendered and the women experienced stereotype threat and isolation, leading to inequity in opportunities for learning [30,31].

Academic heterogeneity can also produce challenges, such as low-ability students relying on high-ability students to complete the task. For example, some studies show that students tend to improve reasoning ability in groups with homogeneity in ability as they needed to interact and find solutions to tasks [32]. These groups likely could not rely on “social loafing” that could have been possible in a group with ability heterogeneity.

There is relatively little information available on heterogeneous group dynamics and outcomes in contexts where members of typically underrepresented groups are more highly represented (e.g., minority-serving institutions). One possibility is that the risk of negative effects from marginalized students being isolated as the only individual with that identity is reduced when the population of students overall is more diverse.

### The present study

In the present study, we test whether students in groups that are strategically assigned to be maximally heterogeneous experience better outcomes than students in groups that are randomly assigned. Many prior studies examining group composition compare student self-selected groups to randomly-formed groups or examine existing variation among groups without an intervention (e.g., [12–14,16]). However, the existence of tools such as the Comprehensive Assessment of Team Member Effectiveness (CATME) SMARTER Teamwork [32–34] tool enables instructors to strategically form groups, an alternative to both random assignment and self-assortment. CATME is a system of online tools designed to help instructors manage student teams effectively. One of the available tools, which we use for the current study, is a team-maker, which sorts students into teams based on their responses to a survey and criteria that instructors select. In this study, we seek to explore whether strategically creating heterogeneous groups might result in positive benefits for students over randomly-assigned groups. Our study furthers the conversation about optimal group composition in two key ways.

First, we consider both types of heterogeneity – cognitive and identity – in combination as “maximally heterogeneous.” Based on prior work indicating benefits of both cognitive heterogeneity and identity heterogeneity, we hypothesized that combining both types of heterogeneity would yield positive attitudes towards learning, superior group performance, and reduced amounts of conflict during group work.

Second, we examine the effects of heterogeneity in a minority-serving institution where a group that is typically underrepresented is well-represented. Specifically, this study was conducted at a Hispanic-Serving Institution, which had 29% enrollment of Hispanic students and more broadly speaking 39% enrollment of students with underrepresented racial/ethnic backgrounds during the year the study took place. We hypothesize that the risk of marginalized students having negative social experiences from isolation will be mitigated due to their relatively high representation and therefore students in heterogeneous groups will experience improved outcomes.

## Methods

We used a quasi-experimental design to assess how lab group composition affected students’ group work performance, grades, incidence of conflict, teamwork satisfaction, and perceptions of collaborative learning. In an introductory biology course sequence for majors, we designated half the lab sections to have groups assigned to maximize heterogeneity in demographic characteristics and prior preparation (using CATME) and the other half of the lab sections had groups assigned to be random with respect to these characteristics. All human subjects research procedures were reviewed and determined exempt by Texas Tech University’s IRB (IRB2022–799). The study was conducted from August 2022 through May 2023. Informed consent was not obtained because all procedures were commonly accepted classroom practices. The data underlying the results presented in the study are available in the Open Science Framework repository at https://osf.io/9uyzh/?view_only=28a0850e0bf64359be985b3c1aac4d85.

### Study setting and participants

We conducted this study at a single large, public, Very High Research Activity (R1) Hispanic-serving institution in the United States. The introductory biology course sequence for majors integrates both lecture and lab in the same course and spans two semesters. The course in the fall covers cellular biology, genetics, and energetics and the course in the spring covers evolution, ecology, and physiology (only one of the courses is available each semester). There are no pre-requisites for either course in the sequence, and students could take the courses in either order, though one is only offered in each semester (more details below). Two of the authors are the instructors of record for this course sequence: ASN is the lab coordinator who supervises the graduate Teaching Assistants (TAs) who are the lab instructors but does not directly instruct students and LBL is the overall course instructor of record who teaches the lecture sections but is not directly involved in lab instruction.

During each semester, groups worked on all lab activities together. Mid-semester, groups planned and executed an independent investigation project, *i.e.,* an experiment based on previous lab activities that was developed and designed by groups themselves with guidance from TAs. Students were not provided explicit guidance on group work, but before working on the lab report, they completed a group contract in which they outlined expectations of contributions from each group member and designated an internal deadline for revisions prior to submission. At the end of the semester, groups submitted a collaborative lab report that the team submitted as a single report and all group members receive the same grade. However, students completed a peer review in which they indicated whether all group mates contributed equally and fairly. If group members reported that one group member did not contribute sufficiently to the report, that individual was or could have been assigned a lower grade per the TA’s discretion. In both semesters, the lab report was worth 8% of the lab grade (in turn, the lab grade was worth 30% of the overall course grade).

Many students take both courses in sequence, but students may take just one if they only require one for a major in a different department (e.g., agriculture, kinesiology, bioengineering) or transfer credit to replace one of the courses. Thus, the population of students in the two courses typically contains a large amount of overlap but is not exactly the same population. In our sample, 528 participants took both courses, 268 participants enrolled in the fall only (not the spring), and 100 participants enrolled in the spring only (not the fall). Due to the large but incomplete overlap, we implemented the quasi-experimental procedures in both semesters but treated them separately and considered the spring a replication attempt. In the fall, there were 798 students enrolled in the course across 36 lab sections (~24 students in each section) taught by 18 graduate Teaching Assistants (TAs; each TA teaches two lab sections). In the spring, there were 628 students enrolled in the course across 28 lab sections taught by 14 TAs. The demographic diversity of the participants is described in Table 1. Note that the number of participants is lower than the total enrollment in the course due to some students choosing to not participate in the research.

### Group assignments

Each TA taught two lab sections; in one of their sections, they created maximally heterogeneous groups and in the other section created randomly-assigned groups. All groups were composed of 4 members at the start of the semester and groups were retained throughout the semester. If a group member dropped the course, the group remained intact with 3 members. In this academic year, no group had more than one member drop the course.

Maximally heterogeneous groups were created using CATME. To create groups using CATME, students completed a short survey at the start of each semester, providing information about their identities (*i.e.,* race, gender, generation in college), prior preparation (GPA), weekly schedule, and future goals (complete survey text available in S1 File). To create maximally heterogeneous groups, CATME settings were assigned to create groups dissimilar in race, gender, generation in college, and GPA. Schedules were assigned to be similar to facilitate groups finding common time to work together on lab group projects. Random groups were assigned either by TAs before the first lab session or during the first lab session (*e.g.*, by randomly handing out papers with group numbers on them).

We examined the structures of groups in sections where groups were created by CATME and randomly assigned to check that the intervention changed the pattern of group structures in the sections (Table 2). For each demographic characteristic, we characterized groups as being homogenous (*e.g.*, being all white students or all women), having a student with a marginalized identity isolated in a group (*e.g.*, 3 white students and a Black student or 3 men and a woman), or being mixed (*e.g.*, 2 white students and 2 Hispanic students or 2 men and 2 women). We observed clear differences in the group makeup across the different strategies for creating groups. In both semesters, the sections where groups were created to be maximally heterogeneous had fewer homogenous groups and more groups that were mixed and with isolated URM students or isolated women. This provided confirmation that using CATME created groups that were noticeably different in composition from when groups were assigned at random.

**Table 2:** Group structures.

Group structures^1^	Fall 2022		Spring 2023	
	**Heterogeneous**	**Random**	**Heterogeneous**	**Random**
Total number of groups in semester	108	107	83	81
**Group Race and Ethnicity** ^2^				
Homogenous non-URM	2	9	6	7
Isolated URM	30	21	33	17
Isolated non-URM	11	9	12	11
Mixed	43	21	25	15
Homogenous URM	1	5	3	3
NA^3^	21	42	4	28
**Group Binary Gender** ^4^				
All men	0	14	1	11
All women	29	32	20	21
Isolated woman	21	16	21	8
Isolated man	39	28	24	24
Mixed	12	16	14	16
Isolated gender minoritized identity	0	0	2	1
NA	7	1	1	0
**Group Generation**				
Isolated first generation	59	24	50	13
Isolated continuing generation	6	9	3	2
Mixed	25	9	21	12
All first generation	0	2	0	3
All continuing generation	6	9	8	15
NA	12	54	1	36

^1^Data for creating maximally heterogeneous groups were obtained from the CATME survey and data for randomly-assigned groups were obtained from institutional data and end of semester survey responses since these students did not complete the CATME survey.

^2^Underrepresented Minorities (URM) was defined following the National Science Foundation’s definition which includes Blacks or African Americans, Hispanics or Latinos, and American Indians or Alaska Natives.

^3^NA = Missing data for one or more group members prevented us from identifying the group makeup.

^4^Available gender data is binary due to the limitations of the data collection tools. The author team acknowledges the limitations of considering binary gender in analyses and regrets that the data we have do not reflect students with different gender identities.

Group structures of maximally heterogenous and randomly assigned groups across fall and spring semesters.

### Data collection

#### Group conflict.

We collected peer evaluations from all students. The evaluations asked students to provide percentages for quality of contribution and for commitment to the project for each of their group members. If they assigned a low percent for any group member, they were asked to provide justification and explanation for their low rating. This was completed as an in-lab task but not graded for any course points. We qualitatively assessed these evaluations and identified a group having “conflict” if at least one group member indicated that one or more of their group members did not contribute in an equal manner to their project and/or report. In this event, TAs reached out to group members to discuss the situation, attempting to mediate the conflict, and if needed escalated the situation to the lab coordinator. In cases of extreme conflict, the lab coordinator held mediation meetings with the concerned students. Grades were assigned as a group regardless of conflict/s reported.

#### Report and lab grades.

At the end of each semester, the research team collected the final lab report grade and overall lab grade for each student. Lab reports were graded with a rubric that was common across all lab sections and all lab sections included the same number and type of assignments with the same weighting for calculating the total lab grade. The lab report grading rubric consisted of 64 checkpoints; the grade was the percentage of checkpoints that were satisfied in the report (see S2 File for the full check list reproduced with permission from Stipes Publishing). The rubric was designed to assess skills we aimed for students to develop through the independent investigation and lab report activity. The rubric included items about experimental design (e.g., “Was a control treatment and/or baseline measurement included when appropriate?”), clarity of study description (e.g., “Are the research problem and hypothesis clearly stated?”), presentation of results (e.g., “Have the most effective graphical or tabular formats been chosen to present important data?”), discussion of results (e.g., “Are the conclusions supported by the data?”), report elements (e.g., “Do citations appear wherever authors make statement of scientific results that were not part of the authors’ current research?”), and writing quality (e.g., “Have misspellings been eliminated?”).

It is important to note that lab report grades and final lab grades are not fully independent of each other as the lab report grades contribute about 8% to the overall lab grade. We opted to use both the overall lab grade and the lab report grade as outcomes in this study as they capture slightly different performance outcomes. The lab report grade is the more proximal measure of group work performance since the lab report was a collaborative assignment and all lab members received the same grade. In contrast, the overall lab grade consisted of both collaborative assignment grades and grades from individual quizzes and assignments. Thus, the overall lab grade captures individuals’ overall performance in the lab and the extent to which their lab group experience may have also impacted their individual performance.

#### Teamwork satisfaction and perceptions of collaborative learning.

We distributed a post-semester survey to all students that included measures of teamwork satisfaction, perceptions of collaborative learning, and demographic questions. Teamwork satisfaction was measured with 4 items (e.g., “Looking back at the lab, I am satisfied with our teamwork project.”) previously developed and used by Liu and colleagues [35]. There was no prior evidence of validity discussed or presented, but we judged that the content of the items was appropriate (it was developed for use with university students) and they did present evidence of acceptable reliability (Cronbach’s alpha = 0.7). Perceptions of collaborative learning was measured with 7 items (e.g., “Collaborative learning in my group was effective.”) previously developed and used by [36]. They created their scale for undergraduates by adapting multiple pre-existing scales and presented evidence of validity based on internal structure as well as evidence of reliability. The full text of items in the survey is available in S1 File. We received 1,152 responses from the end of semester survey (n = 640 in the fall and n = 512 in the spring), which represents more than three-fourths of the total number of students enrolled in the courses (80.8% response rate). The authors have accounted for students who attended both courses included within the study, and therefore the final dataset included responses from 986 unique respondents.

### Data analysis

Our analysis strategy treats our two semesters as a replication test of the same experiment. We decided to proceed with analyzing data from the two semesters separately for a variety of reasons, including that some students were in both classes, the course content was different (so the samples were not easily combinable), and our hypothesis testing procedure included interactions and we thought our sample size would not support three-way interactions with semester. Therefore, we analyze data from the two semesters separately and qualitatively compare the robustness of findings across both semesters.

#### Assessing evidence of validity for latent variables.

We used confirmatory factor analysis (CFA) to assess evidence of validity based on internal structure. We fit an initial CFA in both datasets to assess the hypothesized measurement model and found unacceptable fit in both semesters. We made iterative data-driven and theory-driven revisions to the measures in an attempt to achieve an acceptable measurement model (see S3 File for detailed methods and results). We arrived at a measurement model for perceptions of collaborative learning that had acceptable fit in the spring and borderline fit in the fall. However, we were unable to obtain satisfactory evidence of measurement invariance across the two types of group formation in either semester (see S3 File for more details on this analysis and results). Therefore, we were not able to analyze how group formation influenced students’ teamwork satisfaction or perceptions of collaborative learning. In other words, the students in heterogeneous and randomly-assigned groups responded to the measures of teamwork satisfaction and perceptions of collaborative learning in fundamentally different ways that made them non-comparable. This could suggest that something about the way groups are formed influenced how students experience the phenomena of teamwork satisfaction and perceptions of collaborative learning. We were unable to explore this further due to the non-invariance across these groups. This is a hypothesis worth pursuing in the future when evaluating strategies for group formation.

#### Model selection.

We followed a 3-step model selection process as described in Theobald et al. [37] using the lme4 package in R version 4.3.1 [38,39]. Briefly, we first determined the random effects structure by fitting a series of models to determine which random effect structure best represents the data. We tested TA, section, and group as random effects because of the nested nature of our data (more details below). To do this, we fit 8 models to test the random effects structure with the most complex model we planned to fit [40]. This allows us to determine the best fitting model considering that random effect structures impact the estimates of fixed effects, and vice versa [37]. The model we fit while determining the best random effect structure included an interaction between group formation strategy (heterogeneous vs. random) and first-generation status. We chose first-generation status because this was the group identity which had the greatest number of minoritized students in our sample. We subset the data to only use complete cases. For all model selection procedures, we compared the fit of models using Akaike’s Information Criterion (AIC) values and selected the best fit model as one with the lowest AIC. If the difference in AIC was less than 2, we considered the fit to be equivalent and favored the simpler, more parsimonious model [37]. We report all models tested during the model selection process and AIC values in S4 File.

We started by testing the model structure including all possible random effects. In our study, we identified 3 possible random effects: effects caused by grouping at the student level within their lab groups (group), at the section level (section) and at the teaching assistant level since each TA taught two sections (TA). We fit these random effects with random intercepts, not slopes because there is no reason to suspect that there is a differential impact of these factors on the outcome [37]. Then we proceeded to assess the best model structure by including different combinations of the three identified random effects (group + section, section + TA, group + TA), followed by determining their individual contribution to the model (group only, TA only, section only), and finally assessed a model in their absence (null model). We used restricted maximum likelihood as our model estimator which is the default selection in the lme4 package in R. We identified the best random effects structure for each of our outcomes separately.

After selecting the best random effects structure of our analysis models, we then selected the best fitting fixed effect structure. We followed a backwards design selection as described by Theobald and colleagues [37]; we started with a model containing all our fixed effects (group formation method and identity variables and an interaction between these) and the best fitting random effects structure, subsetting the data for each demographic variable and using complete cases only. We then successively removed each fixed effect from the model, starting with the interaction term, and assessed change in the AIC values. For these models, we did not use restricted maximum likelihood (i.e., REML = F) [37]. We determined fixed effects structures for our demographic variables of gender, race/ethnicity, and generation in college using separate sets of models because we did not collect enough data to support models with three- or four-way interactions. Finally, we interpreted the best fitting models (i.e., those with the lowest AIC) for each of our demographics separately for the two semesters.

## Results

**Fig 1:**
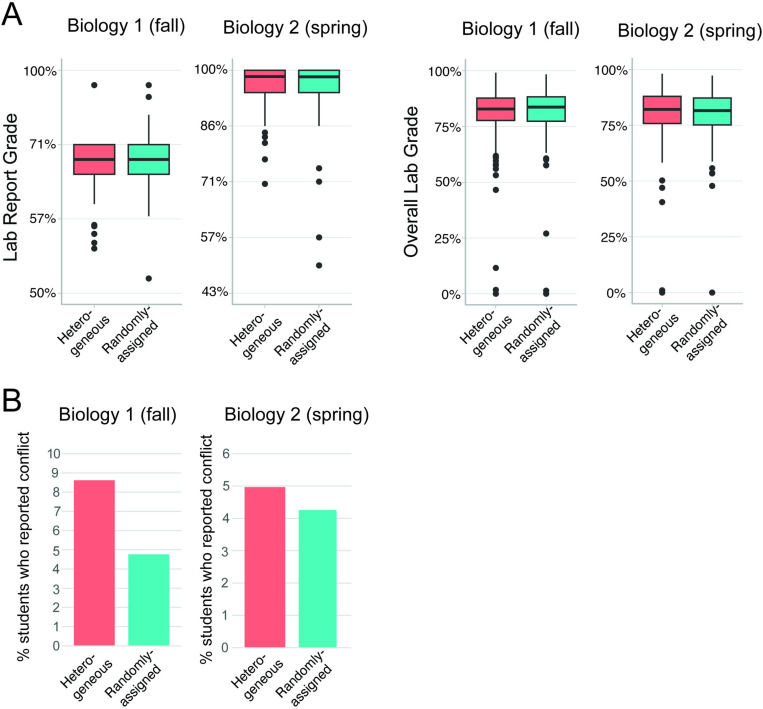
Lab report and final lab grades (A) and incidence of conflicts (B) for students in maximally heterogeneous groups and randomly-assigned groups in both courses.

Our model comparisons indicated that group formation did not consistently impact students’ grades (Table 3; Fig 1). In the fall, group formation had no impact on students’ grades, but in the spring, creating heterogeneous groups eliminated racial disparity in lab report grades (but did not impact overall lab grades). We were unable to analyze reports of conflict due to models failing to converge, likely due to the low incidence of reports of conflict. We were also not able to analyze the latent variables we intended to measure (teamwork satisfaction and perceptions of collaborative learning) because we were unable to achieve acceptable internal structure and measurement invariance across the two types of group formation (see methods).

**Table 3:** Parameter estimates for best-fitting models.

Outcome	Course	Best Model	Intercept	Group Formation	Identity	Formation*Identity
Lab Report	Bio1^1^^,^^2^	Null Model^1^^,^^2^	23.82 (0.21)			
	Bio2^1^^,^^3^	Race*Formation^1^^,^^3^	33.82 (0.24)	-0.06 (0.25) (Formation: Heterogeneous)	-0.60 (0.18) (URM: True)	0.61 (0.25)
Overall Lab Grade	Bio1^1^^,^^4^	Model with demographics^1^^,^^4^	83.77 (0.57)^5^			
	Bio2^1^^,^^4^	Model with demographics^1^^,^^4^	81.59 (0.85)^5^			

^1^ Random effect structure included a random slope for Group ID and TA

^2^ Model selection procedure additionally tested binary gender, race/ethnicity, and first generation status and the null model was preferred in all of these models.

^3^ Model selection procedure additionally tested binary gender and first generation status; in those cases the null model was preferred.

^4^ Model selection procedure additionally tested binary gender, race/ethnicity, and first generation status; the best fitting model in each case included the demographic indicator but not group formation indicator or the interaction

^5^ Intercept for null model shown; see S4 File for intercepts for models that included demographic indicator.

### Preliminary exploratory analyses

First, we conducted basic preliminary data exploration. We visually inspected the data by graphing the lab report grades, overall lab grades, and frequency of students reporting conflict disaggregated by group formation method for both courses (Fig 1). A simple visual inspection suggested that there were not any obvious differences across the two modes of group formation in students’ lab report grades or overall lab grades (Fig 1A). Conflicts were very infrequently reported, ranging from 4–9% of students being in groups that reported conflict (Fig 1B).

### Hypothesis testing model selection results

We tested a series of models to identify which variables best explained the variance in each outcome in each semester. The final, best fitting models for both outcomes (lab report grade and final lab grade) are presented in Table 3. For all of the models, the best random effect structure included the group and TA variables, but not the section variable. For all but one model, the group formation fixed effect was absent from the final, best-fitting model. This means that whether groups were formed randomly or strategically to be maximally heterogeneous had no impact on the outcomes.

Parameter estimates are shown with standard error in parentheses. For indicator variables, the reference group is indicated in parentheses.

### Model results for lab report grade

For lab report grade in biology 1 (fall semester course), the null model was the most parsimonious, best fitting model, which indicates that we were unable to significantly detect meaningful effects of the hypothesized fixed effects (gender, race/ethnicity, generation in college, nor group formation; Table 3). However, in biology 2 (the spring semester course), the model with an interaction between group formation method (heterogeneous vs. random) and URM fit the best. The retention of the interaction term in the best-fitting model revealed that the lab report grades for URM students (m = 33.22) were lower than non-URM students (m = 33.82) in the randomly-assigned groups only. In the heterogenous groups, URM students’ lab report grades were improved (m = 33.75) whereas non-URM students’ lab report grades were unchanged (m = 33.74), which removed performance disparity. It is important to note the caveat that this finding was only in biology 2 and that we could not detect any effect of race/ethnicity in biology 1, thus this result was not robust and could be spurious.

### Model results for overall lab grade

In both courses, the group formation variable (random vs. heterogeneous) was not included in the final, best-fitting model, meaning that lab group formation method did not impact students’ overall lab grades in either course.

In both courses, the final model included all 3 demographics as fixed effects (but not group formation or any interaction effects), indicating that gender, racial/ethnic identity, and generation in college all impacted students’ overall lab grades. These results repeated trends known from prior literature that students with minoritized social identities experience worse academic outcomes. Final lab grades were lower for students identifying as women than men in both biology 1 (m_women_ = 83.05; m_men_ = 85.22) and in biology 2 (m_women_ = 80.87; m_men_ = 82.96). Similarly, URM students scored 3.17 points less than non-URM counterparts in biology 1 and 2.09 points less in biology 2. Finally, students who were the first in their family to attend college (first generation) scored 1.78 points less than their counterparts whose family members have attended college (continuing generation) in biology 1 and 3.66 points less in biology 2.

## Discussion

We failed to find consistent evidence that maximally heterogeneous lab groups improved group performance. In three of the four models, we found no effect of group formation on students’ grades. In one semester, creating groups to be maximally heterogeneous eliminated the racial disparity that was present in randomly-created groups in the grades on the collaborative lab report, but this effect did not carry through to impact the overall lab grade. This finding was promising, but was not robust across semesters or outcomes.

We collected data to assess the impact of group formation on other outcomes, but were unable to analyze them. Fortunately, conflict in groups was reported so infrequently across both semesters and treatments (<9% of students) that we were unable to estimate models predicting incidence of conflict. One possibility is that the groups were highly functional regardless of how they were formed, which yielded comparably successful outcomes.

We also hypothesized that group structure would impact students’ group work satisfaction and perceptions of collaborative learning, but we were unable to assess these outcomes due to measurement nonequivalence between the two treatments of group formation. However, the measurement nonequivalence itself points to an interesting implication: something about the two different strategies of forming the groups affected how students interpreted and responded to the items measuring teamwork satisfaction and perceptions of collaborative learning. It seems that students in randomly-assigned and maximally heterogeneous groups had substantially different groupwork experiences in a way that our outcome measures were not able to capture. A fruitful next step would be to conduct cognitive interviews with students from different types of groups to gain insight into how they interpreted the items and look for systematic differences across group formation strategy. Future work could also take an exploratory, qualitative approach to examining how the experiences of students differ across group composition.

Our null results have multiple possible explanations. One possibility is that the hypothesized benefits of maximally heterogeneous groups were not realized in this context. Another possibility is that the hypothesized benefits were realized, but were offset by challenges that emerged in maximally heterogeneous groups, such as “soloing” students with marginalized identities [27]. One consequence of maximally heterogeneous group composition is that students with underrepresented identities, which are often also marginalized, are isolated within a group of others with more overrepresented identities. Prior research indicates that students with marginalized identities can have negative experiences working with peers in groups [27]. For example, women report being not respected and talked over in mixed-gender lab groups in introductory science [41]. More generally speaking, lacking friends in one’s group and being uncomfortable with one’s group mates is associated with poorer learning outcomes [15]. Extensive research suggests that marginalized students can feel uncomfortable during group work. For example, racially marginalized students prefer to listen rather than lead discussions compared to white students during peer discussion [42]. However, in contrast, Asgari, Cardace and Sarvary found that demographic isolation did not impact students’ attitudes about groupwork [13].

If maximally heterogeneous groups created challenges with marginalized students being isolated, we might have observed an increase in reports of conflicts in maximally heterogeneous groups. However, we did not see strong evidence of this in our dataset (Fig 1B). Although we were not able to estimate mixed effects models predicting conflict (likely due to the low incidence across the board), inspection of the raw data does not suggest a robust trend. There were more reports of conflict in maximally heterogeneous groups in the fall (9% in heterogeneous groups vs. 4% in randomly-assigned groups), but no difference in the spring (4–5% in both types of groups).

This study is also limited in that both groups did not complete the CATME survey – only the lab sections assigned to have maximally heterogenous groups completed this survey as part of the group assignment protocol. If completing the survey had some impact on teamwork, then this survey might potentially have been a confounding effect. We also did not consider heterogeneity levels across the two mechanisms of group formation; we only considered group composition to confirm that more groups in the maximally heterogenous condition matched that condition. Future studies could consider investigating this aspect in relation to teamwork dynamics.

Students’ experiences are shaped by their unique, intersecting identities. We were not able to investigate interactions between identities (e.g., race and gender) because our sample size was not large enough to support three-way and four-way interactions. Future studies should investigate intersecting identities of students in classes which may substantially influence students’ experiences and outcomes. Future studies could also determine whether the degree of diversity will impact experiences by including contextual variables such as proportion of group members with a particular membership criterion, and how these might impact the outcome variables. Researchers might also consider providing instruction and guidance on working effectively as a team and study how this influences students’ teamwork satisfaction. Another perspective could be to determine how students interact in a team when they have prior experience with teamwork, relationships with their team members, and the effects of other variables, such as personality and relevant prior knowledge and skills.

## Conclusion

We conducted a quasi-experiment in a large-enrollment introductory biology lab class in which half of the lab sections assigned groups randomly and half assigned groups to be maximally heterogeneous in terms of race, gender, and prior preparation. We did not find consistent evidence that group formation strategy impacted group performance on the lab report or on overall lab grades. In one semester, assigning groups to be maximally heterogeneous eliminated a racial disparity in lab report grades that was present in randomly-assigned groups, but this effect did not translate into changes in overall lab grades and was only observed in one of the two semesters. Conflicts were reported so infrequently that we were not able to fully analyze them, but did not see strong evidence of conflict being more common in one group.

## Supporting information

S1 File Full text of the CATME and post-semester surveys. (DOCX)

S2 File Checklist for lab report grading. Reproduced with permission from Stipes Publishing, the publisher of the lab manual used for this course.
(PDF)

S3 File Supplemental information on latent variable analysis. (DOCX)

S4 File Supplemental information on model selection. (DOCX)
