# Supplementary material for: Strategically creating maximally heterogeneous lab groups did not improve group performance in an introductory biology lab class
Source: PLoS One. 2025 May 15;20(5):e0323799. doi: 10.1371/journal.pone.0323799 (PMC12080782; doi:10.1371/journal.pone.0323799)
Supplement: S1 File — (DOCX) [file pone.0323799.s001.docx]

**S1 File.** Full text of the CATME and post-semester surveys.

**CATME survey items.** CATME-based survey distributed to undergraduate students in CATME designated sections and used for group assignment. Q1-Q3 were obtained from the CATME question manager and the remaining questions were designed by the authors.

1. Biological sex:
   - Response options: Male, Female
2. Race: Please indicate the racial/ethnic group with which you most identify:
   - Response options: White, Hispanic, Black, Native, Asian, Other, Declined
3. GPA: Your overall GPA is _______.
4. Schedule: Please check the times that you are in class, at work or practice and are busy and unavailable for group work.
5. Generation: Which of the following accurately describes your family’s education?
   - Continuing generation: At least one of my parent(s)/guardian(s) has earned a 4-year college degree.
   - First generation: None of my parent(s)/guardian(s) has earned a 4-year college degree.
6. Area of interest in biology: Which area of biology interests you the most?
   - Response options: Ecology, Human biology, Computational biology, Genetics, Microbiology, Cell/molecular, Neurobiology/neuroscience, Botany/plant sciences, Kinesiology, Environmental, Zoology, Anatomy and Physiology, Immunology, Wildlife biology and conservation, Entomology, Aquatic biology, Agricultural biology, Veterinary Biology, Other
7. Future goals: What are your future career goals?
   - Response options: Pre-med, Pre-nursing, Graduate school, Veterinary school, Physician’s assistant, Nurse practitioner, Pharmaceuticals, Physical therapist, Other

**Post-term survey items**. Post-semester survey administered to all enrolled undergraduates in the lab course.

**Student Evaluations of TA:** Designed by departmental faculty and approved by department. Response scale: 1-Strongly Disagree, 2-Disagree, 3-Slightly Disagree, 4-Slightly Agree, 5-Agree, 6-Strongly Agree.

1. My TA was prepared for class.
2. My TA demonstrated a knowledge of the subject.
3. My TA conveyed major points and concepts.
4. My TA presented content at a level which I easily understood.
5. My TA fostered participation by asking questions and comments from students.
6. My TA communicated clearly.
7. MY TA was responsive to my emails/requests to meet outside of class.
8. My TA treated students with respect and fairness.
9. The graded assignments were relevant to my learning.
10. My TA promptly returned assignments.
11. My TA stimulated my interest in the subject.
12. My TA helped when I encountered experimental difficulties.
13. Overall, my TA was an effective instructor.
14. The lab activities helped my understanding of the scientific process
15. The concepts and skills I learned in this lab course will be valuable for my future education or my career.

**Teamwork satisfaction:** Liu, Majuka, Lee (2008)

Response scale: 1-Strongly disagree, 2-Disagree, 3-Neutral, 4-Agree, 5-Strongly agree.

1. Looking back at the lab, I am satisfied with our teamwork project.
2. I think I learned many meaningful lessons throughout lab group projects.
3. Overall, I believe that whole teamwork process of our lab group is valuable to driving us toward team goals.
4. Overall, I believe that lab group came up with the best solutions as we expected.

**Perception of collaborative learning:** So and Brush (2008) collected validity based on internal structure for a population of students pursuing higher education.

Response scale: 1-Strongly disagree, 2-Disagree, 3-Neutral, 4-Agree, 5-Strongly agree.

1. I felt part of a learning community in my group.
2. I actively exchanged my ideas with group members.
3. I was able to develop new skills and knowledge from other members in my group.
4. I was able to develop problem solving skills through peer collaboration.
5. Collaborative learning in my group was effective.
6. Collaborative learning in my group was time-consuming.
7. Overall, I am satisfied with my collaborative learning experience in this course.

**Demographic items**

1. Generation: Which of the following accurately describes your family’s education?
   - Continuing generation: At least one of my parent(s)/guardian(s) has earned a 4-year college degree.
   - First generation: None of my parent(s)/guardian(s) has earned a 4-year college degree.
   - Prefer not to respond
2. Classification: Which of the following accurately describes your current classification at the university?
   - First year
   - Second year
   - Third year
   - Fourth year
   - Other (please specify)
3. Major: Which of the following describes your subject major at the university?
   - Life Sciences
   - Chemistry
   - Physics
   - Engineering
   - Math
   - Non-STEM
   - Other (please specify)
4. Race/Ethnicity: With which race(s) and ethnicity/ies do you identify? Select all that apply:
   - Black (African American, African, or Caribbean)
   - East Asian (e.g., China and Japan)
   - South Asian (e.g., the Indian sub-continent)
   - Southeast Asian (e.g., Vietnam)
   - Latinx or Hispanic
   - Middle Eastern or North African
   - Native American or Alaskan Native
   - Native Hawaiian or Pacific Islander
   - White
   - Other, please specify
   - Prefer not to respond
5. Gender: What gender do you identify as?
   - Man
   - Woman
   - Non-binary
   - Not listed above, please specify
   - Prefer not to respond
