# Supplementary material for: Strategically creating maximally heterogeneous lab groups did not improve group performance in an introductory biology lab class
Source: PLoS One. 2025 May 15;20(5):e0323799. doi: 10.1371/journal.pone.0323799 (PMC12080782; doi:10.1371/journal.pone.0323799)
Supplement: S2 File — Reproduced with permission from Stipes Publishing, the publisher of the lab manual used for this course. (PDF) [file pone.0323799.s002.pdf]

Checklist for Scientific Research Paper

BIOL 1403/1404

Texas Tech University

(You should be able to answer yes to all these questions.)

Satisfactory	Unsatisfactory	
		**Experimental Design**
❒	❒	Was the experiment chosen for the paper based on a theory-based, testable hypothesis capable of producing conclusive results?
❒	❒	Were quantitative data generated capable of analysis by *t*-test?
❒	❒	Were replications maximized within time and resource limitations?
❒	❒	Was maximized by reasonable control of variables?
❒	❒	Was a control treatment and/or baseline measurement (i.e., blank for spec calibration) included when appropriate?
❒	❒	Were errors like untested assumptions and sampling bias prevented?
❒	❒	Was it conducted and reported with integrity and objectivity?
		**Title**
❒	❒	Does the title accurately describe the contents of the paper?
❒	❒	Is the title specific enough (i.e., give enough information)?
❒	❒	Is the title concise enough (i.e., give too much information)?
		**Abstract**
❒	❒	Is it ten sentences, or fewer, in length?
❒	❒	Does it contain a statement of the research problem?
❒	❒	Does it contain a statement of the hypothesis?
❒	❒	Does it contain a statement of the methods used?
❒	❒	Does it contain a statement of the chief results?
❒	❒	Does it contain a statement of the conclusion(s)?
		**Introduction**
❒	❒	Are the research problem and hypothesis clearly stated?
❒	❒	Is the rationale for the experiment included?
❒	❒	Is the history/background included, and in sufficient detail?
❒	❒	Are citations used properly, rather than footnotes?
		**Materials and Methods**
❒	❒	Are materials described in sufficient detail for repetition, if necessary?
❒	❒	Are methods described in sufficient detail for repetition, if necessary?
❒	❒	If the lab manual was used as a reference, was it paraphrased and properly cited?
❒	❒	Is the method of statistical analysis mentioned here?
❒	❒	Is this section free of results or discussion?

Satisfactory	Unsatisfactory	
❒	❒	Has the author avoided listing the materials?
❒	❒	Has the author avoided making the methods appear as a series of directions, as though this were a lab manual rather than a research paper where methods are properly a historical description?
		**Results**
❒	❒	Have all the results been described in the text of the results section?
❒	❒	Have only the most relevant data been selected to be reported as tables and figures?
❒	❒	Have all figures and tables been cited in the text of the results section?
❒	❒	Have the most effective graphical or tabular formats been chosen to present important data?
❒	❒	Can figures and tables be understood without having to refer to the text?
❒	❒	Are figures properly titled and captioned (below figure)?
❒	❒	Are tables properly titled and captioned (above table)?
❒	❒	Where applicable, does dependent variable appear on vertical axis, independent variable on horizontal axis?
❒	❒	Are tables and figures numbered independently, and are they numbered according to the sequence in which they are cited in the text?
❒	❒	Has a t-test been correctly performed? Has it been reported and interpreted correctly?
❒	❒	Is the word “significantly” always used in association with a statistical level of certainty?
❒	❒	Is the results section well-organized?
❒	❒	Is the results section free of methodology or interpretation?
		**Discussion**
❒	❒	Has the author avoided claiming that results “prove” or “disprove” the hypothesis?
❒	❒	Are the conclusions supported by the data?
❒	❒	Have all possible conclusions been drawn from the data, i.e., have all the data’s repercussions been discussed?
❒	❒	Where applicable, have anomalies been reported and addressed?
❒	❒	Have assumptions been identified and justified?
❒	❒	Where applicable, has the relationship between these and previous findings been discussed?
❒	❒	Are possibilities for further research presented, and are they realistic in the context of this course?
❒	❒	Is the discussion section well organized?
❒	❒	Is the discussion section free of methodology or results?

Acknowledgments

❒	❒	Where applicable, are intellectual contributors (outside the research group) identified?
❒	❒	Are sources of borrowed equipment/supplies/organisms identified?
❒	❒	Where applicable, are sources of funding identified?
❒	❒	Where applicable, are contributors of donated services (i.e., typing, reviewing, editing, etc.) identified?

Literature Cited

❒	❒	Do all references cited also appear in the text?
❒	❒	Are all citations consistently presented in the same format?
❒	❒	Are citations presented in an established format?
❒	❒	Do citations appear wherever authors make statement of scientific results that were not part of the authors' current research?

General

❒	❒	Is the paper consistently written in the past tense?
❒	❒	Are all foreign words (inc. scientific binomials) italicized or underlined?
❒	❒	Have typos been eliminated?
❒	❒	Have misspellings been eliminated?
❒	❒	Does the entire paper exhibit stylistic consistency?
❒	❒	If applicable, have editorial comments on the draft version been addressed?
❒	❒	Did the authors use citations rather than footnotes?
