# Supplementary material for: Strategically creating maximally heterogeneous lab groups did not improve group performance in an introductory biology lab class
Source: PLoS One. 2025 May 15;20(5):e0323799. doi: 10.1371/journal.pone.0323799 (PMC12080782; doi:10.1371/journal.pone.0323799)
Supplement: S3 File — (DOCX) [file pone.0323799.s003.docx]

**S3 File.** Supplemental Information on latent variable analyses

We used confirmatory factor analysis (CFA) to assess evidence of validity based on internal structure. We fit all models using robust maximum likelihood estimator to account for non-normality of the data and report the robust fit statistics.

We first fit an initial CFA to both datasets to assess the hypothesized measurement model. We found an unacceptable fit in both semesters (Supplemental Table 1). Examining parameter estimates in the CFA of the hypothesized model revealed that item 6 within perceptions of collaborative learning exhibited poor fit (only item with loading <0.4). We reviewed the text of the item: “Collaborative learning with my group was time consuming.” We determined that this item was dissimilar from the other items and did not represent the construct well because (1) effective learning is often time consuming and (2) amount of time spent does not necessarily relate to students’ perceptions of the utility of collaborative learning. Thus, we removed this item from the measure and re-fit the CFA (Revised model 1). The fit of the first revised model was unacceptable in both semesters (Supplemental Table 1).

We examined the modification indices for both models which indicated that several of the most impactful modifications would be adding covariances between items on separate latent variables and adding loadings of items onto the latent variable it is not hypothesized to belong to. We also observed that the covariance between the two latent variables was extremely high (> 0.85) in both models. Based on these results, we decided to test whether the latent variables were indeed separate constructs. We fit a unidimensional model with all items loading onto a single latent variable, but we found worse fit than when they were estimated as separate latent variables in both semesters (Supplemental Table 1 unidimensional model).

We then fit separate models for teamwork satisfaction and perceptions of collaborative learning to further diagnose the fit issues. Both latent variables exhibited unsatisfactory fit in both semesters (Supplemental Table 1). Teamwork satisfaction had worse fit and modification indices suggested multiple adjustments would be needed, so we opted to proceed with attempting to improve the perceptions of collaborative learning model only. Modification indices for both semesters suggested the addition of a residual covariance between items 5 and 7. We fit a revised collaborative learning model with this residual covariance added (Supplemental Table 1, collaborative learning revised model). The revised model had acceptable fit in the spring and borderline acceptable fit in the fall (RMSEA was >0.1, but confidence interval overlaps 0.08). The modification indices on the models in the fall and spring did not agree on the next modification, so we decided not to make further modifications and proceed with testing for measurement invariance.

Supplemental Table 1. fit metrics of confirmatory factor analysis models.

| Model | Semester | CFI | TLI | SRMR | RMSEA | RMSEA 90% confidence interval |
| --- | --- | --- | --- | --- | --- | --- |
| Hypothesized model | Fall | 0.945 | 0.930 | 0.035 | 0.116 | 0.102 - 0.131 |
|  | Spring | 0.970 | 0.962 | 0.028 | 0.082 | 0.064 - 0.101 |
| Revised model 1 | Fall | 0.951 | 0.936 | 0.030 | 0.122 | 0.105 - 0.140 |
|  | Spring | 0.972 | 0.963 | 0.027 | 0.090 | 0.068 - 0.112 |
| Unidimensional model | Fall | 0.921 | 0.899 | 0.040 | 0.153 | 0.137 - 0.171 |
|  | Spring | 0.898 | 0.869 | 0.054 | 0.169 | 0.149 - 0.169 |
| Collaborative learning only | Fall | 0.958 | 0.931 | 0.028 | 0.168 | 0.136 - 0.202 |
|  | Spring | 0.980 | 0.967 | 0.023 | 0.110 | 0.064 - 0.158 |
| Teamwork only | Fall | 0.983 | 0.949 | 0.021 | 0.153 | 0.085 - 0.231 |
|  | Spring | 0.967 | 0.902 | 0.027 | 0.225 | 0.160 - 0.298 |
| Collaborative learning revised | Fall | 0.983 | 0.969 | 0.021 | 0.113 | 0.077 - 0.151 |
|  | Spring | 0.991 | 0.983 | 0.018 | 0.079 | 0.020 - 0.079 |

Our goal was to compare the teamwork satisfaction and perceptions of collaborative learning of students in heterogenous groups to students in randomly-assigned groups. Comparing latent constructs across groups requires first establishing measurement invariance to ensure that something about the way groups were assigned did not systematically affect how students would respond to these survey items. We were unable to obtain satisfactory measurement invariance, with or without the iterative revisions we made to both measurement models.

We first conducted an omnibus test of measurement invariance by comparing a model where all parameters estimates were constrained to be equal across the groups (fully constrained model) to a model where all parameters for each group were freely estimated (free model). Equivalent fit of the constrained and free models would demonstrate measurement invariance. However, we found that the constrained model was a worse fit than the free model in both semesters (Supplemental Table 2). This result necessitated investigating the cause of the non-invariance.

The first step is configural invariance, that the pattern of items loading onto latent factors is invariant across the groups. Acceptable fit of the fully free model would demonstrate configural invariance. In the fall, the fit is borderline acceptable, and in the spring, the free model has acceptable fit.

The second step is metric invariance, that the magnitude of the loadings are invariant across the groups. We assessed metric invariance by comparing the fully free model to a model with only the loadings constrained across groups (the metric model). However, in both semesters, the metric model was a worse fit than the free model. Therefore, we fail to establish metric invariance. This means we cannot proceed comparing the perceptions of collaborative learning of students in the two types of groups.

Supplemental Table 2: Model fit statistics of measurement invariance models.

| Semester | Model | CFI | TLI | SRMR | RMSEA | RMSEA 90% confidence interval |
| --- | --- | --- | --- | --- | --- | --- |
| Fall | Fully constrained | 0.964 | 0.969 | 0.270 | 0.110 | 0.082 - 0.138 |
|  | Free | 0.984 | 0.971 | 0.021 | 0.106 | 0.065 - 0.148 |
|  | Metric | 0.988 | 0.982 | 0.026 | 0.083 | 0.041 - 0.122 |
| Spring | Fully constrained | 0.994 | 0.995 | 0.106 | 0.043 | 0 - 0.089 |
|  | Free | 0.993 | 0.987 | 0.018 | 0.070 | 0 - 0.127 |
|  | Metric | 0.993 | 0.991 | 0.040 | 0.059 | 0 - 0.111 |

**S3 File.** Supplemental information on model selection

**Step 1: Determining random effects structure for models:**

**Supplemental Table 3. Lab report grade random effects model selection for fall 2022***.* Model fit indices for alternative random effects structures models. These models are predicting lab report grade as the outcome, include group formation and generation indicators as fixed effects, and all random effects reported were specified as random intercepts, not random slopes.

| **Model #** | **Random effects in the model (F22)** | **AIC** | **Delta AIC from best** |
| --- | --- | --- | --- |
| 1a | Group + Section + TA | 2201.2 | 2.0 |
| 2a | Section + Group | 2228.4 | 29.2 |
| 3a | TA + Section | 2452.2 | 253.0 |
| **4a** | **TA + Group** | **2199.2** | **0.0** |
| 5a | Group | 2228.5 | 29.3 |
| 6a | Section | 2483.7 | 284.5 |
| 7a | TA | 2450.2 | 251.0 |
| Null.a | No random effects | 2224.2 | 250. |

**Supplemental Table 4. Lab report grade random effects model selection for spring 2023***.* Model fit indices for alternative random effects structures models. These models are predicting lab report grade as the outcome, include group formation and generation indicators as fixed effects, and all random effects reported were specified as random intercepts, not random slopes.

| **Model #** | **Random effects in the model (S23)** | **AIC** | **Delta AIC from best** |
| --- | --- | --- | --- |
| 1b | Group + Section + TA | 2346.4 | 4.7 |
| 2b | Section + Group | 2348.1 | 6.4 |
| 3b | TA + Section | 2402.1 | 60.4 |
| **4b** | **TA + Group** | **2344.4** | **0.0** |
| 5b | Group | 2346.7 | 5.0 |
| 6b | Section | 2404.3 | 62.6 |
| 7b | TA | 2400.4 | 58.7 |
| Null.b | No random effects | 2341.7 | 2.7 |

**Supplemental Table 5. Lab overall grade random effects model selection for fall 2022.** Model fit indices for alternative random effects structures models. These models are predicting lab overall grade as the outcome, include group formation and generation indicators as fixed effects, and all random effects reported were specified as random intercepts, not random slopes.

| **Model #** | **Random effects in the model (S23)** | **AIC** | **Delta AIC from best** |
| --- | --- | --- | --- |
| 1c | Group + Section + TA | 4502.1 | 2.0 |
| 2c | Section + Group | 4505.0 | 4.9 |
| 3c | TA + Section | 4514.9 | 14.8 |
| **4c** | **TA + Group** | **4500.1** | **0.0** |
| 5c | Group | 4508.2 | 8.1 |
| 6c | Section | 4520.3 | 20.1 |
| 7c | TA | 4512.9 | 12.8 |
| Null.c | No random effects | 4512.1 | 12.0 |

**Supplemental Table 6. Lab overall grade random effects model selection for spring 2023.** Model fit indices for alternative random effects structures models. These models are predicting lab overall grade as the outcome, include group formation and generation indicators as fixed effects, and all random effects reported were specified as random intercepts, not random slopes.

| **Model #** | **Random effects in the model (S23)** | **AIC** | **Delta AIC from best** |
| --- | --- | --- | --- |
| 1d | Group + Section + TA | 3815.8 | 1.6 |
| 2d | Section + Group | 3818.4 | 4.2 |
| 3d | TA + Section | 3820.5 | 6.3 |
| **4d** | **TA + Group** | **3814.2** | **0.0** |
| 5d | Group | 3827.9 | 13.7 |
| 6d | Section | 3822.9 | 8.7 |
| 7d | TA | 3820.7 | 6.5 |
| Null.d | No random effects | 3846.1 | 31.9 |

**Step 2: Determining fixed effect structure for models:**

**Supplemental Table 7. Lab report grade fixed effects model selection for fall 2022.** Random effect structure is TA & Group.

| **Demographic** | **Model #** | **Fixed effects included** | **AIC** | **Delta AIC from best by demographic** |
| --- | --- | --- | --- | --- |
| **Gender:**  Total n = 644  Woman = 432  Man = 212 | 1aGenderF22 | CATME * Gender | 2176.8 | 5.8 |
|  | 1bGenderF22 | CATME + Gender | 2174.9 | 3.9 |
|  | 1cGenderF22 | CATME | 2172.9 | 0.9 |
|  | **1GenderF22null** | **Null (no fixed effects, only random effects)** | **2171.0** | **0.0** |
| **Race/ethnicity:**  Total n = 644  White/Asian = 380  URM = 264 | 1aRaceF22 | CATME * URM | 2176.7 | 5.7 |
|  | 1bRaceF22 | CATME + URM | 2174.8 | 3.8 |
|  | 1dRaceF22 | URM | 2172.9 | 1.9 |
|  | **1RaceF22null** | **Null (no fixed effects, only random effects)** | **2171.0** | **0.0** |
| **Generation in college:**  Total n = 644  First Generation = 210  Continuing Generation = 434 | 1aGenF22 | CATME * Generation | 2174.9 | 3.9 |
|  | 1bGenF22 | CATME + Generation | 2173.7 | 2.7 |
|  | 1dGenF22 | Generation | 2171.8 | 0.8 |
|  | **1GenF22null** | **Null (no fixed effects, only random effects)** | **2171.0** | **0.0** |

**Supplemental Table 8. Lab report grade fixed effects model selection for spring 2023.** Random effect structure is TA & Group.

| **Demographic** | **Model #** | **Fixed effects included** | **AIC** | **Delta AIC from best by demographic** |
| --- | --- | --- | --- | --- |
| **Gender:**  Total n = 530  Woman = 346  Man = 184 | 2aGenderS23 | CATME * Gender | 2000.9 | 4.7 |
|  | 2bGenderS23 | CATME + Gender | 1999.5 | 3.3 |
|  | 2cGenderS23 | CATME | 1997.5 | 1.3 |
|  | **2GenderS23null** | **Null (no fixed effects, only random effects)** | **1996.2** | **0.0** |
| **Race/ethnicity:**  Total n = 530  White/Asian = 324  URM = 206 | **2aRaceS23** | **CATME * URM** | **1991.1** | **0.0** |
|  | 2bRaceS23 | CATME + URM | 1994.8 | 3.7 |
|  | 2dRaceS23 | URM | 1993.3 | 2.2 |
|  | 2RaceS23null | Null (no fixed effects, only random effects) | 1996.2 | 5.1 |
| **Generation in college:**  Total n = 530  First Generation = 170  Continuing Generation = 360 | 2aGenS23 | CATME * Generation | 1999.2 | 3.0 |
|  | 2bGenS23 | CATME + Generation | 1998.2 | 1.4 |
|  | 2dGenS23 | Generation | 1996.8 | 0.6 |
|  | **2GenS23null** | **Null (no fixed effects, only random effects)** | **1996.2** | **0.0** |

**Supplemental Table 9. Lab overall grade fixed effects model selection for fall 2022.** Random effect structure is TA & Group.

| **Demographic** | **Model #** | **Fixed effects included** | **AIC** | **Delta AIC from best by demographic** |
| --- | --- | --- | --- | --- |
| **Gender:**  Total n = 644  Woman = 432  Man = 212 | 3aGenderF22 | CATME * Gender | 4410.5 | 2.6 |
|  | 3bGenderF22 | CATME + Gender | 4409.7 | 1.8 |
|  | **3dGenderF22** | **Gender** | **4407.9** | **0.0** |
|  | 3GenderF22null | Null (no fixed effects, only random effects) | 4417.8 | 9.9 |
| **Race/ethnicity:**  Total n = 644  White/Asian = 380  URM = 264 | 3aRaceF22 | CATME * URM | 4393.1 | 3.8 |
|  | 3bRaceF22 | CATME + URM | 4391.2 | 1.9 |
|  | **3dRaceF22** | **URM** | **4389.3** | **0.0** |
|  | 3RaceF22null | Null (no fixed effects, only random effects) | 4417.8 | 28.5 |
| **Generation in college:**  Total n = 644  First Generation = 210  Continuing Generation = 434 | 3aGenF22 | CATME * Generation | 4415.2 | 3.9 |
|  | 3bGenF22 | CATME + Generation | 4413.2 | 1.9 |
|  | **3dGenF22** | **Generation** | **4411.3** | **0.0** |
|  | 3GenF22null | Null (no fixed effects, only random effects) | 4417.8 | 6.5 |

**Supplemental Table 10. Lab final grade fixed effects model selection for spring 2023.** Random effect structure is TA & Group.

| **Demographic** | **Model #** | **Fixed effects included** | **AIC** | **Delta AIC from best by demographic** |
| --- | --- | --- | --- | --- |
| **Gender:**  Total n = 530  Woman = 346  Man = 184 | 4aGenderS23 | CATME * Gender | 3733.9 | 1.6 |
|  | 4bGenderS23 | CATME + Gender | 3731.9 | 0.4 |
|  | **4dGenderS23** | **Gender** | **3732.3** | **0.0** |
|  | 4GenderS23null | Null (no fixed effects, only random effects) | 3738.2 | 5.9 |
| **Race/ethnicity:**  Total n = 530  White/Asian = 324  URM = 206 | 4aRaceS23 | CATME * URM | 3726.0 | 2.3 |
|  | 4bRaceS23 | CATME + URM | 3724.3 | 0.6 |
|  | **4dRaceS23** | **URM** | **3723.7** | **0.0** |
|  | 4RaceS23null | Null (no fixed effects, only random effects) | 3738.2 | 14.5 |
| **Generation in college:**  Total n = 530  First Generation = 360  Continuing Generation = 170 | 4aGenS23 | CATME * Generation | 3718.4 | 2.8 |
|  | 4bGenS23 | CATME + Generation | 3716.4 | 0.8 |
|  | **4dGenS23** | **Generation** | **3715.6** | **0.0** |
|  | 4GenS23null | Null (no fixed effects, only random effects) | 3738.2 | 22.6 |

**Step 3: Final model results**

**Supplemental Table 11. Lab report grade final model results for fall 2022 for each demographic variable.**

| **Model** | **Intercept (SE)** | **CATME** | **Demographic** | **CATME*demographic** |
| --- | --- | --- | --- | --- |
| Gender: Bio1_Report ~ 1 + (1\|Group_ID_F22) + (1\|TA_F22) | 23.82 (0.21) |  |  |  |
| Race/Ethnicity: Bio1_Report ~ 1 + (1\|Group_ID_F22) + (1\|TA_F22) | 23.82 (0.21) |  |  |  |
| Generation: Bio1_Report ~ 1 + (1\|Group_ID_F22) + (1\|TA_F22) | 23.82 (0.21) |  |  |  |

**Supplemental Table 12. Lab report grade final model results for spring 2023 for each demographic variable.**

| **Model** | **Intercept (SE)** | **CATME** | **Demographic** | **CATME*demographic** |
| --- | --- | --- | --- | --- |
| Gender: Bio2_Report ~ 1 + (1\|Group_ID_S23) + (1\|TA_S23) | 33.67 (0.19) |  |  |  |
| Race/Ethnicity: Bio2_Report ~ CATME_S23*URM + (1\|Group_ID_S23) + (1\|TA_S23) | 33.82 (0.24) | -0.06 (0.25)  (CATME: True) | -0.60 (0.18)  (URM: True) | 0.61 (0.25) |
| Generation: Bio2_Report ~ 1 + (1\|Group_ID_S23) + (1\|TA_S23) | 33.67 (0.19) |  |  |  |

**Supplemental Table 13. Lab overall grade final model results for fall 2022 for each demographic variable.**

| **Model** | **Intercept (SE)** | **CATME** | **Demographic** | **CATME*demographic** |
| --- | --- | --- | --- | --- |
| Gender: Bio1_Final_percent ~ Gender + (1\|Group_ID_F22) + (1\|TA_F22) | 85.22 (0.69) |  | -2.17 (0.62)  (Gender: Woman) |  |
| Race/Ethnicity: Bio1_Final_percent ~ URM + (1\|Group_ID_F22) + (1\|TA_F22) | 85.08 (0.59) |  | -3.17 (0.57)  (URM: True) |  |
| Generation: Bio1_Final_percent ~ Generation + (1\|Group_ID_F22) + (1\|TA_F22) | 84.36 (0.58) |  | -1.78 (0.60)  (First generation) |  |

**Supplemental Table 14. Lab overall grade final model results for spring 2023 for each demographic variable.**

| **Model** | **Intercept (SE)** | **CATME** | **Demographic** | **CATME*demographic** |
| --- | --- | --- | --- | --- |
| Gender: Bio2_Final_percent ~ Gender + (1\|Group_ID_S23) + (1\|TA_S23) | 82.96 (0.98) |  | -2.09 (0.75)  (Gender: Woman) |  |
| Race/Ethnicity: Bio2_Final_percent ~ URM + (1\|Group_ID_S23) + (1\|TA_S23) | 82.70 (0.89) |  | -2.89 (0.70)  (URM: True) |  |
| Generation: Bio2_Final_percent ~ Generation + (1\|Group_ID_S23) + (1\|TA_S23) | 82.79 (0.84) |  | -3.66 (0.73)  (First generation) |  |
