# Supplementary material for: Strategically creating maximally heterogeneous lab groups did not improve group performance in an introductory biology lab class
Source: PLoS One. 2025 May 15;20(5):e0323799. doi: 10.1371/journal.pone.0323799 (PMC12080782; doi:10.1371/journal.pone.0323799)
Supplement: S4 File — (DOCX) [file pone.0323799.s004.docx]

**S4 File.** Supplemental information on model selection

**Step 1: Determining random effects structure for models:**

**Supplemental Table 3. Lab report grade random effects model selection for fall 2022***.* Model fit indices for alternative random effects structures models. These models are predicting lab report grade as the outcome, include group formation and generation indicators as fixed effects, and all random effects reported were specified as random intercepts, not random slopes.

| **Model #** | **Random effects in the model (F22)** | **AIC** | **Delta AIC from best** |
| --- | --- | --- | --- |
| 1a | Group + Section + TA | 2201.2 | 2.0 |
| 2a | Section + Group | 2228.4 | 29.2 |
| 3a | TA + Section | 2452.2 | 253.0 |
| **4a** | **TA + Group** | **2199.2** | **0.0** |
| 5a | Group | 2228.5 | 29.3 |
| 6a | Section | 2483.7 | 284.5 |
| 7a | TA | 2450.2 | 251.0 |
| Null.a | No random effects | 2224.2 | 250. |

**Supplemental Table 4. Lab report grade random effects model selection for spring 2023***.* Model fit indices for alternative random effects structures models. These models are predicting lab report grade as the outcome, include group formation and generation indicators as fixed effects, and all random effects reported were specified as random intercepts, not random slopes.

| **Model #** | **Random effects in the model (S23)** | **AIC** | **Delta AIC from best** |
| --- | --- | --- | --- |
| 1b | Group + Section + TA | 2346.4 | 4.7 |
| 2b | Section + Group | 2348.1 | 6.4 |
| 3b | TA + Section | 2402.1 | 60.4 |
| **4b** | **TA + Group** | **2344.4** | **0.0** |
| 5b | Group | 2346.7 | 5.0 |
| 6b | Section | 2404.3 | 62.6 |
| 7b | TA | 2400.4 | 58.7 |
| Null.b | No random effects | 2341.7 | 2.7 |

**Supplemental Table 5. Lab overall grade random effects model selection for fall 2022.** Model fit indices for alternative random effects structures models. These models are predicting lab overall grade as the outcome, include group formation and generation indicators as fixed effects, and all random effects reported were specified as random intercepts, not random slopes.

| **Model #** | **Random effects in the model (S23)** | **AIC** | **Delta AIC from best** |
| --- | --- | --- | --- |
| 1c | Group + Section + TA | 4502.1 | 2.0 |
| 2c | Section + Group | 4505.0 | 4.9 |
| 3c | TA + Section | 4514.9 | 14.8 |
| **4c** | **TA + Group** | **4500.1** | **0.0** |
| 5c | Group | 4508.2 | 8.1 |
| 6c | Section | 4520.3 | 20.1 |
| 7c | TA | 4512.9 | 12.8 |
| Null.c | No random effects | 4512.1 | 12.0 |

**Supplemental Table 6. Lab overall grade random effects model selection for spring 2023.** Model fit indices for alternative random effects structures models. These models are predicting lab overall grade as the outcome, include group formation and generation indicators as fixed effects, and all random effects reported were specified as random intercepts, not random slopes.

| **Model #** | **Random effects in the model (S23)** | **AIC** | **Delta AIC from best** |
| --- | --- | --- | --- |
| 1d | Group + Section + TA | 3815.8 | 1.6 |
| 2d | Section + Group | 3818.4 | 4.2 |
| 3d | TA + Section | 3820.5 | 6.3 |
| **4d** | **TA + Group** | **3814.2** | **0.0** |
| 5d | Group | 3827.9 | 13.7 |
| 6d | Section | 3822.9 | 8.7 |
| 7d | TA | 3820.7 | 6.5 |
| Null.d | No random effects | 3846.1 | 31.9 |

**Step 2: Determining fixed effect structure for models:**

**Supplemental Table 7. Lab report grade fixed effects model selection for fall 2022.** Random effect structure is TA & Group.

| **Demographic** | **Model #** | **Fixed effects included** | **AIC** | **Delta AIC from best by demographic** |
| --- | --- | --- | --- | --- |
| **Gender:**  Total n = 644  Woman = 432  Man = 212 | 1aGenderF22 | CATME * Gender | 2176.8 | 5.8 |
|  | 1bGenderF22 | CATME + Gender | 2174.9 | 3.9 |
|  | 1cGenderF22 | CATME | 2172.9 | 0.9 |
|  | **1GenderF22null** | **Null (no fixed effects, only random effects)** | **2171.0** | **0.0** |
| **Race/ethnicity:**  Total n = 644  White/Asian = 380  URM = 264 | 1aRaceF22 | CATME * URM | 2176.7 | 5.7 |
|  | 1bRaceF22 | CATME + URM | 2174.8 | 3.8 |
|  | 1dRaceF22 | URM | 2172.9 | 1.9 |
|  | **1RaceF22null** | **Null (no fixed effects, only random effects)** | **2171.0** | **0.0** |
| **Generation in college:**  Total n = 644  First Generation = 210  Continuing Generation = 434 | 1aGenF22 | CATME * Generation | 2174.9 | 3.9 |
|  | 1bGenF22 | CATME + Generation | 2173.7 | 2.7 |
|  | 1dGenF22 | Generation | 2171.8 | 0.8 |
|  | **1GenF22null** | **Null (no fixed effects, only random effects)** | **2171.0** | **0.0** |

**Supplemental Table 8. Lab report grade fixed effects model selection for spring 2023.** Random effect structure is TA & Group.

| **Demographic** | **Model #** | **Fixed effects included** | **AIC** | **Delta AIC from best by demographic** |
| --- | --- | --- | --- | --- |
| **Gender:**  Total n = 530  Woman = 346  Man = 184 | 2aGenderS23 | CATME * Gender | 2000.9 | 4.7 |
|  | 2bGenderS23 | CATME + Gender | 1999.5 | 3.3 |
|  | 2cGenderS23 | CATME | 1997.5 | 1.3 |
|  | **2GenderS23null** | **Null (no fixed effects, only random effects)** | **1996.2** | **0.0** |
| **Race/ethnicity:**  Total n = 530  White/Asian = 324  URM = 206 | **2aRaceS23** | **CATME * URM** | **1991.1** | **0.0** |
|  | 2bRaceS23 | CATME + URM | 1994.8 | 3.7 |
|  | 2dRaceS23 | URM | 1993.3 | 2.2 |
|  | 2RaceS23null | Null (no fixed effects, only random effects) | 1996.2 | 5.1 |
| **Generation in college:**  Total n = 530  First Generation = 170  Continuing Generation = 360 | 2aGenS23 | CATME * Generation | 1999.2 | 3.0 |
|  | 2bGenS23 | CATME + Generation | 1998.2 | 1.4 |
|  | 2dGenS23 | Generation | 1996.8 | 0.6 |
|  | **2GenS23null** | **Null (no fixed effects, only random effects)** | **1996.2** | **0.0** |

**Supplemental Table 9. Lab overall grade fixed effects model selection for fall 2022.** Random effect structure is TA & Group.

| **Demographic** | **Model #** | **Fixed effects included** | **AIC** | **Delta AIC from best by demographic** |
| --- | --- | --- | --- | --- |
| **Gender:**  Total n = 644  Woman = 432  Man = 212 | 3aGenderF22 | CATME * Gender | 4410.5 | 2.6 |
|  | 3bGenderF22 | CATME + Gender | 4409.7 | 1.8 |
|  | **3dGenderF22** | **Gender** | **4407.9** | **0.0** |
|  | 3GenderF22null | Null (no fixed effects, only random effects) | 4417.8 | 9.9 |
| **Race/ethnicity:**  Total n = 644  White/Asian = 380  URM = 264 | 3aRaceF22 | CATME * URM | 4393.1 | 3.8 |
|  | 3bRaceF22 | CATME + URM | 4391.2 | 1.9 |
|  | **3dRaceF22** | **URM** | **4389.3** | **0.0** |
|  | 3RaceF22null | Null (no fixed effects, only random effects) | 4417.8 | 28.5 |
| **Generation in college:**  Total n = 644  First Generation = 210  Continuing Generation = 434 | 3aGenF22 | CATME * Generation | 4415.2 | 3.9 |
|  | 3bGenF22 | CATME + Generation | 4413.2 | 1.9 |
|  | **3dGenF22** | **Generation** | **4411.3** | **0.0** |
|  | 3GenF22null | Null (no fixed effects, only random effects) | 4417.8 | 6.5 |

**Supplemental Table 10. Lab final grade fixed effects model selection for spring 2023.** Random effect structure is TA & Group.

| **Demographic** | **Model #** | **Fixed effects included** | **AIC** | **Delta AIC from best by demographic** |
| --- | --- | --- | --- | --- |
| **Gender:**  Total n = 530  Woman = 346  Man = 184 | 4aGenderS23 | CATME * Gender | 3733.9 | 1.6 |
|  | 4bGenderS23 | CATME + Gender | 3731.9 | 0.4 |
|  | **4dGenderS23** | **Gender** | **3732.3** | **0.0** |
|  | 4GenderS23null | Null (no fixed effects, only random effects) | 3738.2 | 5.9 |
| **Race/ethnicity:**  Total n = 530  White/Asian = 324  URM = 206 | 4aRaceS23 | CATME * URM | 3726.0 | 2.3 |
|  | 4bRaceS23 | CATME + URM | 3724.3 | 0.6 |
|  | **4dRaceS23** | **URM** | **3723.7** | **0.0** |
|  | 4RaceS23null | Null (no fixed effects, only random effects) | 3738.2 | 14.5 |
| **Generation in college:**  Total n = 530  First Generation = 360  Continuing Generation = 170 | 4aGenS23 | CATME * Generation | 3718.4 | 2.8 |
|  | 4bGenS23 | CATME + Generation | 3716.4 | 0.8 |
|  | **4dGenS23** | **Generation** | **3715.6** | **0.0** |
|  | 4GenS23null | Null (no fixed effects, only random effects) | 3738.2 | 22.6 |

**Step 3: Final model results**

**Supplemental Table 11. Lab report grade final model results for fall 2022 for each demographic variable.**

| **Model** | **Intercept (SE)** | **CATME** | **Demographic** | **CATME*demographic** |
| --- | --- | --- | --- | --- |
| Gender: Bio1_Report ~ 1 + (1\|Group_ID_F22) + (1\|TA_F22) | 23.82 (0.21) |  |  |  |
| Race/Ethnicity: Bio1_Report ~ 1 + (1\|Group_ID_F22) + (1\|TA_F22) | 23.82 (0.21) |  |  |  |
| Generation: Bio1_Report ~ 1 + (1\|Group_ID_F22) + (1\|TA_F22) | 23.82 (0.21) |  |  |  |

**Supplemental Table 12. Lab report grade final model results for spring 2023 for each demographic variable.**

| **Model** | **Intercept (SE)** | **CATME** | **Demographic** | **CATME*demographic** |
| --- | --- | --- | --- | --- |
| Gender: Bio2_Report ~ 1 + (1\|Group_ID_S23) + (1\|TA_S23) | 33.67 (0.19) |  |  |  |
| Race/Ethnicity: Bio2_Report ~ CATME_S23*URM + (1\|Group_ID_S23) + (1\|TA_S23) | 33.82 (0.24) | -0.06 (0.25)  (CATME: True) | -0.60 (0.18)  (URM: True) | 0.61 (0.25) |
| Generation: Bio2_Report ~ 1 + (1\|Group_ID_S23) + (1\|TA_S23) | 33.67 (0.19) |  |  |  |

**Supplemental Table 13. Lab overall grade final model results for fall 2022 for each demographic variable.**

| **Model** | **Intercept (SE)** | **CATME** | **Demographic** | **CATME*demographic** |
| --- | --- | --- | --- | --- |
| Gender: Bio1_Final_percent ~ Gender + (1\|Group_ID_F22) + (1\|TA_F22) | 85.22 (0.69) |  | -2.17 (0.62)  (Gender: Woman) |  |
| Race/Ethnicity: Bio1_Final_percent ~ URM + (1\|Group_ID_F22) + (1\|TA_F22) | 85.08 (0.59) |  | -3.17 (0.57)  (URM: True) |  |
| Generation: Bio1_Final_percent ~ Generation + (1\|Group_ID_F22) + (1\|TA_F22) | 84.36 (0.58) |  | -1.78 (0.60)  (First generation) |  |

**Supplemental Table 14. Lab overall grade final model results for spring 2023 for each demographic variable.**

| **Model** | **Intercept (SE)** | **CATME** | **Demographic** | **CATME*demographic** |
| --- | --- | --- | --- | --- |
| Gender: Bio2_Final_percent ~ Gender + (1\|Group_ID_S23) + (1\|TA_S23) | 82.96 (0.98) |  | -2.09 (0.75)  (Gender: Woman) |  |
| Race/Ethnicity: Bio2_Final_percent ~ URM + (1\|Group_ID_S23) + (1\|TA_S23) | 82.70 (0.89) |  | -2.89 (0.70)  (URM: True) |  |
| Generation: Bio2_Final_percent ~ Generation + (1\|Group_ID_S23) + (1\|TA_S23) | 82.79 (0.84) |  | -3.66 (0.73)  (First generation) |  |
